# Short- and Long-Term Effects of LRRK2 on Axon and Dendrite Growth

**DOI:** 10.1371/journal.pone.0061986

**Published:** 2013-04-30

**Authors:** Bryan Sepulveda, Roxana Mesias, Xianting Li, Zhenyu Yue, Deanna L. Benson

**Affiliations:** 1 Department of Neuroscience, Mount Sinai School of Medicine, New York, New York, United States of America; 2 Department of Neurology, Friedman Brain Institute, Mount Sinai School of Medicine, New York, New York, United States of America; 3 Graduate School of Biological Sciences, Mount Sinai School of Medicine, New York, New York, United States of America; University of Nebraska Medical Center, United States of America

## Abstract

Mutations in leucine-rich repeat kinase 2 (*LRRK2*) underlie an autosomal-dominant form of Parkinson's disease (PD) that is clinically indistinguishable from idiopathic PD. The function of LRRK2 is not well understood, but it has become widely accepted that LRRK2 levels or its kinase activity, which is increased by the most commonly observed mutation (G2019S), regulate neurite growth. However, growth has not been measured; it is not known whether mean differences in length correspond to altered rates of growth or retraction, whether axons or dendrites are impacted differentially or whether effects observed are transient or sustained. To address these questions, we compared several developmental milestones in neurons cultured from mice expressing bacterial artificial chromosome transgenes encoding mouse wildtype-LRRK2 or mutant LRRK2-G2019S, *Lrrk2* knockout mice and non-transgenic mice. Over the course of three weeks of development on laminin, the data show a sustained, negative effect of LRRK2-G2019S on dendritic growth and arborization, but counter to expectation, dendrites from *Lrrk2* knockout mice do not elaborate more rapidly. In contrast, young neurons cultured on a slower growth substrate, poly-L-lysine, show significantly reduced axonal and dendritic motility in *Lrrk2* transgenic neurons and significantly increased motility in *Lrrk2* knockout neurons with no significant changes in length. Our findings support that LRRK2 can regulate patterns of axonal and dendritic growth, but they also show that effects vary depending on growth substrate and stage of development. Such predictable changes in motility can be exploited in LRRK2 bioassays and guide exploration of LRRK2 function *in vivo*.

## Introduction

Mutations in the gene encoding leucine-rich repeat kinase 2 (*LRRK2*) account for up to 13% of familial Parkinson's disease (PD) cases. The most common clinical mutation, G2019S, has been reported in almost 7% of familial PD patients with penetrance that is age-dependent–from 21% at age 50 to 81% at age 70 [1], [2], [3]. The structure of LRRK2 suggests that it is multifunctional; it is a 286 kDa protein that contains several protein-protein interaction domains, a kinase domain, and a GTPase [3].The G2019S mutation increases LRRK2 kinase activity [4], [5], [6], [7], suggesting that therapies designed to reduce kinase activity may be relevant for treating PD [8]; however, the biological functions of LRRK2 are poorly understood.

Several efforts have been made to identify predictable effects of altered LRRK2 levels or kinase activity in neurons grown in culture that could be used as bioassays as well as to pursue disease-related phenotypes *in vivo*. Previous work has shown that neurons grown in culture that overexpress LRRK2-G2019S have shorter neurites than non-transgenic neurons, while those cultured from mice with *Lrrk2* deleted (KO) have longer neurites [9], [10], [11], [12]. These data have led many in the field to believe that LRRK2, and the G2019S mutation in particular, inhibits growth. However, there remain several outstanding questions that suggest that this widely accepted interpretation requires closer examination and clarification. Previous studies have measured neurite length in glutamatergic neurons on a single day [9], [10], [11], with differences interpreted as either changes in growth or retraction, measures that require longitudinal observations. Neurites have not been parsed into axons and dendrites [8], [9], [11], [12] so it is not known whether shorter lengths could be unique to axons, dendrites or both. It is also not clear whether changes in neurite length reflect altered levels of LRRK2, LRRK2 mutation or even the means by which LRRK2 is introduced in neurons since *in vivo* observations in bacterial artificial chromosome (BAC) transgenic mouse lines and in LRRK2-KO mice do not exhibit phenotypes consistent with shorter neurites reported *in vitro* [11], [12], [13].

Here we used genetic LRRK2 mouse models to determine whether LRRK2 levels or the G2019S mutation altered the establishment of neural polarity, the elaboration of axons and dendrites, and/or the pattern of axon and dendrite growth over the course of development. Our data support a model in which LRRK2 has a consistent effect on dendrite and axon growth that is best revealed on a slow growth substrate using time-lapse imaging. These data support the use of neurite growth assays as a reflection of LRRK2 actions, but also indicate that such assays need to be utilized with caution.

## Results

### Mouse Models

To test whether LRRK2 levels and mutation affect neuronal development, we cultured neurons from mouse lines that have been described previously. We used BAC transgenic mouse lines that overexpress either mouse wildtype LRRK2 (LRRK2-WT^OE^) or LRRK2 carrying the G2019S mutation (LRRK2-G2019S^OE^) (13). Because the BAC LRRK2 expression is controlled by the endogenous promoter, the spatio-temporal expression of additional LRRK2 expression resembles the endogenous [13], [14], [15], [16]. Additionally, we investigated the effect of a targeted *Lrrk2* deletion in neuronal development with *Lrrk2* knockout (KO; [10]) mice.

### Acquisition of Neuronal Polarity Unchanged by LRRK2-WT^OE^, LRRK2-G2019S^OE^ or KO

Since LRRK2 is suspected to regulate neurite growth, we asked whether the development of neuronal polarity, an event that is defined by the focused growth of a single process that becomes an axon, is altered by LRRK2 levels or mutation. In hippocampus, LRRK2 is expressed in most neurons beginning in late embryonic stages [14], [15], [16], [17], so we used hippocampal neurons grown in culture as a model. We tested for the acquisition of neuronal polarity in three different ways.

Measurement of all neurites at 24 h reveals that 100% of neurons of all four genotypes had grown one neurite that was at least 10 µm longer than all of the others, which previous work has demonstrated is the axon [18].

**Figure 1:**
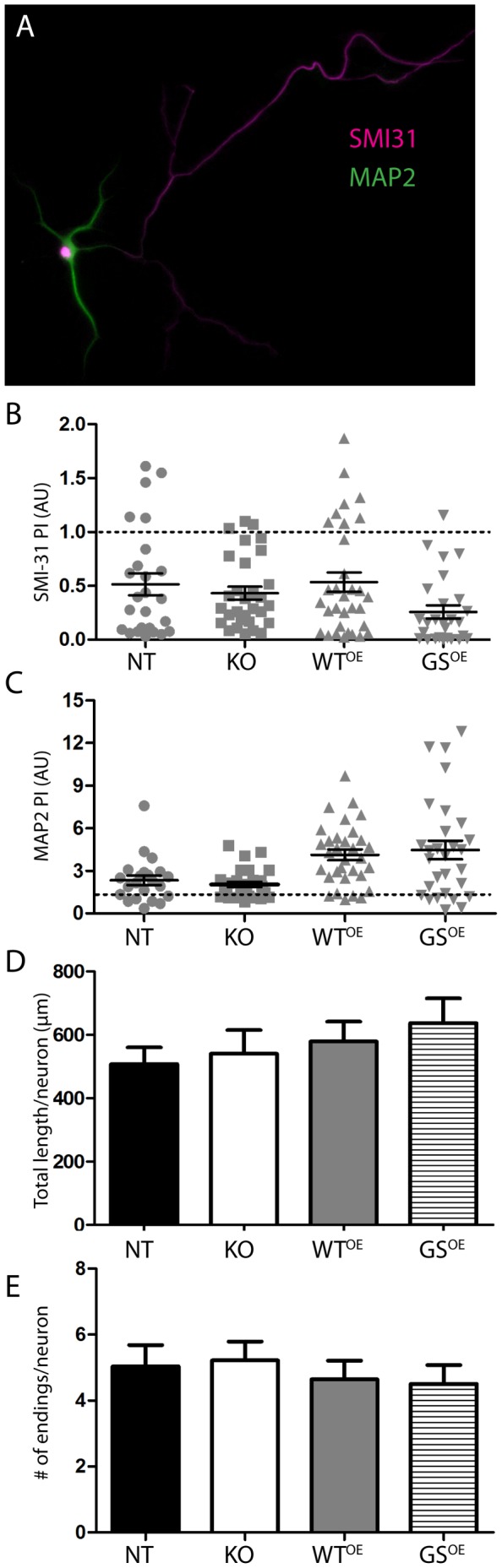
LRRK2 levels or mutation do not affect axonal development or branching. (A) Image of a 3 DIV LRRK2-WT^OE^ transgenic neuron immunolabeled for SMI-31 (magenta) and MAP2 (green) illustrates the normal polarization of proteins achieved in neurons from all genotypes. Scatter plots of polarity indices (PI) calculated for 3 DIV neurons of each genotype for SMI-31 (B) and for MAP2 (C). Mean ± SEM indicated by horizontal line and error bars. At least 10 neurons from two culture preparations each (at least 20 neurons total) were analyzed per genotype. Bar graphs of mean axon lengths (D) and mean number of axon terminal endings (E) measured in fixed 3 DIV neurons (± SEM). At least 10 neurons from at least two culture preparations each (at least 20 neurons total) were traced and measured per genotype.

Polarity can also be defined by the sorting of axonal and dendritic proteins to their respective subcellular domains. To measure this, neurons were grown for 3 days *in vitro* (DIV) and labeled for SMI-31, which concentrates in axons as soon as they are generated and MAP2, which becomes enriched in dendrites and excluded from axons over a slower time course [19], [20]. To compare polarized distribution among genotypes quantitatively, we generated a polarity index (PI) for MAP2 by dividing the immunofluorescence intensity of MAP2 in dendrites by the intensity of MAP2 in axons (i.e. MAP2_dendrite/axon_) and a PI for SMI-31 by dividing the immunofluorescence intensity of SMI-31 in dendrites by the intensity of SMI-31 in axons (i.e. SMI-31_dendrite/axon_) [21] (Fig. 1A–C). An advantage of this assessment is that it is independent of fluorescence intensity levels, which can vary between neurons. Properly polarized neurons have a PI for SMI-31 less than one and for MAP2, greater than one. The data show that axons and dendrites in neurons of all genotypes have appropriately polarized SMI-31 and MAP2 immunolabeling, defined by a ratio of MAP2_dendrite/axon_ greater than 1 (Chi-square, p = 0.128) and SMI-31_dendrite/axon_ less than 1 (Chi-square, p = 0.090).

Since primary hippocampus neurons can abnormally form multiple axons, we asked whether LRRK2 activity had an effect on multiple axon formation [22], [23]. For each genotype, at least 50 neurons in at least two different culture preparations were scored for the presence of multiple axons defined by SMI-31 labeling. Few neurons of any genotype had multiple axons, and there were no significant differences between groups (Chi-square, p = 0.480; NT: 4.49%; KO: 3.45%; LRRK2-WT^OE^: 2.00%; LRRK2-G2019S^OE^: 8.89%).

### Axon Growth and Branching are not altered by LRRK2-WT^OE^, LRRK2-G2019S^OE^ or KO

To determine whether changes in LRRK2 levels or LRRK2 mutation alter total axon length or branching patterns, SMI-31-labeled axons in neurons cultured on laminin from mice of all four genotypes were traced in their entirety at 3 DIV. Neither total axon length, nor numbers of branches, determined by the number of dendritic branch tips, differed significantly between groups (one-way ANOVA; length, p = 0.61; branches, p = 0.83; Fig. 1D, E).

### LRRK2 has Modest Effects on Dendritic Growth and Arborization

**Figure 2:**
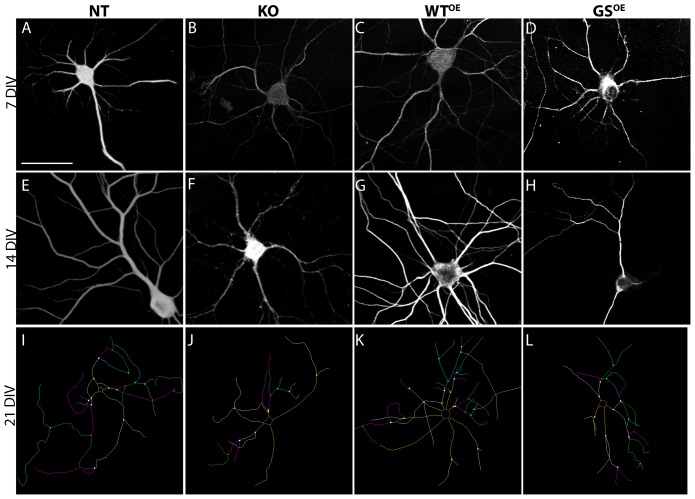
Dendrites from all genotypes grow and arborize over the course of three weeks. Confocal images of MAP2 immunolabeled neurons at 7 DIV (A–D) and 14 DIV (E–H). Magnification bar = 50 µm. These and similar preparations were traced in their entirety using Neurolucida. (I–L) Examples of such tracings are shown for 21 DIV neurons. Color-coding of dendrite branches corresponds to branch order. The maps shown are not to scale, but have been sized to fit the frame.

Previous work suggests that dendrite length can be affected by LRRK2 levels or mutation [24]. To determine whether such changes reflect altered extension or retraction, whether they are transient or sustained, and/or whether differences observed reflect changes in numbers of primary dendrites or dendritic branches, the dendritic arbors in neurons grown on laminin from all four genotypes were traced in their entirety at 3, 5, 7, 10, 14 and 21 DIV (Fig. 2). A power analysis (see Methods) indicated that measurements from 20 neurons per time point is sufficient to detect even a small effect size, and we set this as a minimum.

**Figure 3:**
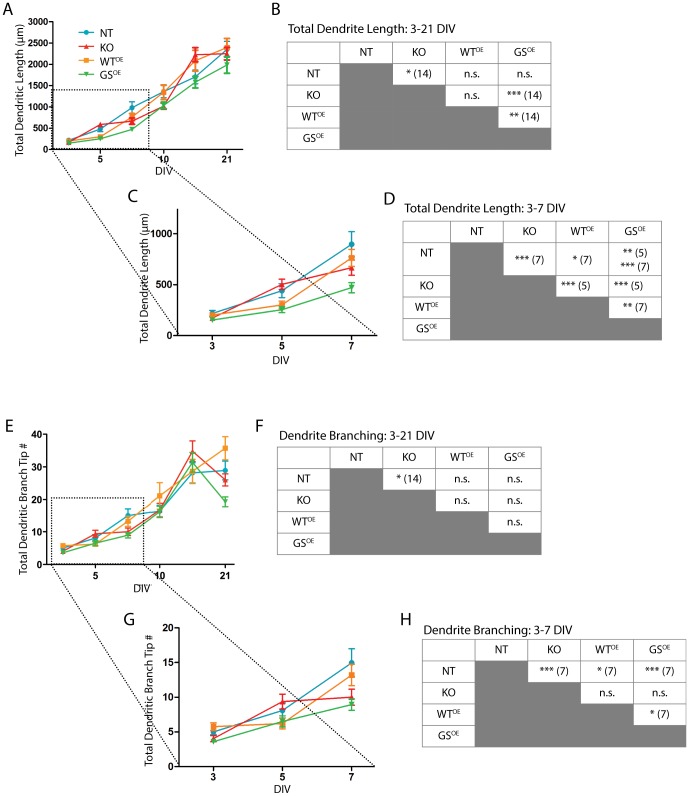
LRRK2-G2019S^OE^ negatively regulates dendritic growth and branching. Line graph (A) plots total dendritic length per neuron over time for neurons of all four genotypes. Data are shown as mean ± SEM. Table (B) shows results of Bonferroni's post-tests, indicating the sources of significant differences identified in a two-way ANOVA (see text). *p<0.05; **p<0.01; ***p<0.001, n.s.  =  not significant. Numbers in parentheses refer to DIV. Line graph (C) is an expanded view of results from the first week of development shown in (A), and the table (D) shows the post hoc analyses from this subset. Line graph (E) plots the total number of dendritic branch points for all genotypes and the table (F) shows the results of Bonferroni's post-tests. The line graph (G) is an expanded view of the data shown in (E) and the table (H) shows the post hoc analyses from the first week of development. At least 10 neurons from at least two culture preparations each (at least 20 neurons total) were traced and measured per genotype per time point assessed.

When total dendritic lengths per neuron are compared, it can be seen that the growth curve of LRRK2-G2019S^OE^ neurons sits below that of all other genotypes (Fig. 3A). Neurons from all genotypes elaborate dendritic arbors over a three week period, but the growth rate for LRRK2-G2019S^OE^ neurons is significantly reduced (two-way ANOVA, age, p<0.0001; genotype, p = 0.0008; Fig. 3A). A post hoc test shows that the differences are greatest at 14 DIV (Fig. 3B).

Several lines of data had led us to predict that KO neurons would display increased dendritic growth relative to all other genotypes (see Introduction). With this in mind, we asked whether growth curves over the first week in culture–a time period that covers most previous studies–differ from later ages. LRRK2-G2019S^OE^ dendrites show significantly reduced growth (two-way ANOVA, p<0.0001 for both genotype and age; Fig. 3C, D). Interestingly, there was a significant increase in KO dendrite growth compared to NT at 5 DIV, but measurements at 3 and 7 DIV showed the reverse with NT dendrites significantly longer than KO.

To determine whether LRRK2 regulates dendritic branching, we compared numbers of primary dendrites and numbers of total branch endings per neuron. There were no significant differences between any of the four genotypes in the number of primary dendrites per neuron (1-way ANOVA, p = 0.64; mean ± SEM of all ages: NT: 4.3±0.4; LRRK2-WT^OE^: 4.5±0.5; KO: 3.9±0.4; LRRK2-G2019S^OE^: 4.0±0.2). Dendrite branch endpoint numbers increase over time and similarly for all genotypes (two-way ANOVA, age, p = 0.001; genotype, p = 0.12; Fig. 3E, F). However, a separate examination of the first week in culture reveals an early effect of genotype on dendrite branching, with LRRK2-G2019S^OE^ neurons having fewer dendritic branches than NT and LRRK2-WT^OE^ neurons (two-way ANOVA, genotype, p = 0.002; time, p<0.0001; Fig. 3G, H). Interestingly, KO and LRRK2-G2019S^OE^ dendrites also have significantly fewer branches than NT dendrites at 7 DIV. However, these effects are not sustained when data from 10–21 DIV are compared separately (two-way ANOVA, genotype, p = 0.33; time, p<0.0001).

We asked whether a change in branching patterns could have contributed to the decrease in dendrite growth observed in KO neurons at 21 DIV. The data show that KO neurons have an exceptionally high number of fifth degree and higher branches at 14 DIV that are lost at 21 DIV (mean ± SEM: 44.9±9.8 branches at 14 DIV; 15.6±3.3 branches at 21 DIV).

Since LRRK2-G2019S^OE^ neurons display more restrained growth rates and slightly diminished branching, and because the LRRK2-G2019S^OE^ mutation has been associated with increased neuron death [6], [9], [25], [26], we asked whether LRRK2-G2019S^OE^ neurons survive as well under our conditions as neurons from other genotypes. At 14 DIV, there were no significant differences in neuron densities between any of the genotypes (one-way ANOVA, p = 0.10).

### Time Lapse Imaging and a Slow Substrate Reveal Clear Effect of LRRK2 on Motility

Previous assessments of neurite length at single time points would have predicted that neurites from LRRK2-G2019S^OE^ neurons grow slower than those in NT neurons, while KO neurites grow faster. Consistent with this prediction, our data show decreased rates of dendritic growth in LRRK2-G2019S^OE^ neurons compared to NT and KO, but there is no change in axonal or dendritic growth rates in KO neurons compared to NT. Thus, we asked whether our experimental conditions could be masking an effect on KO neurons. One possibility is that rapid growth on laminin generated a ceiling effect. Laminin is a natural growth substrate that was chosen based on pilot studies because it best supported the survival and maintenance of mouse neurons in culture. However, poly-L-lysine (PLL), an artificial and much slower substrate, has been used in most previous studies to grow cultured neurons [10], [11]. Another possibility is that neuron-to-neuron variability masks a modest, but consistent effect.

To address these possibilities, time-lapse imaging was used to track growth on PLL in the same neurites over time. Neurons were imaged every 15 minutes for 7.5 h and two parameters were compared: 1) *motility*, defined as the absolute value of movement–extension or retraction–in any direction from the starting point; and 2) *growth* (extension minus retraction).

**Figure 4:**
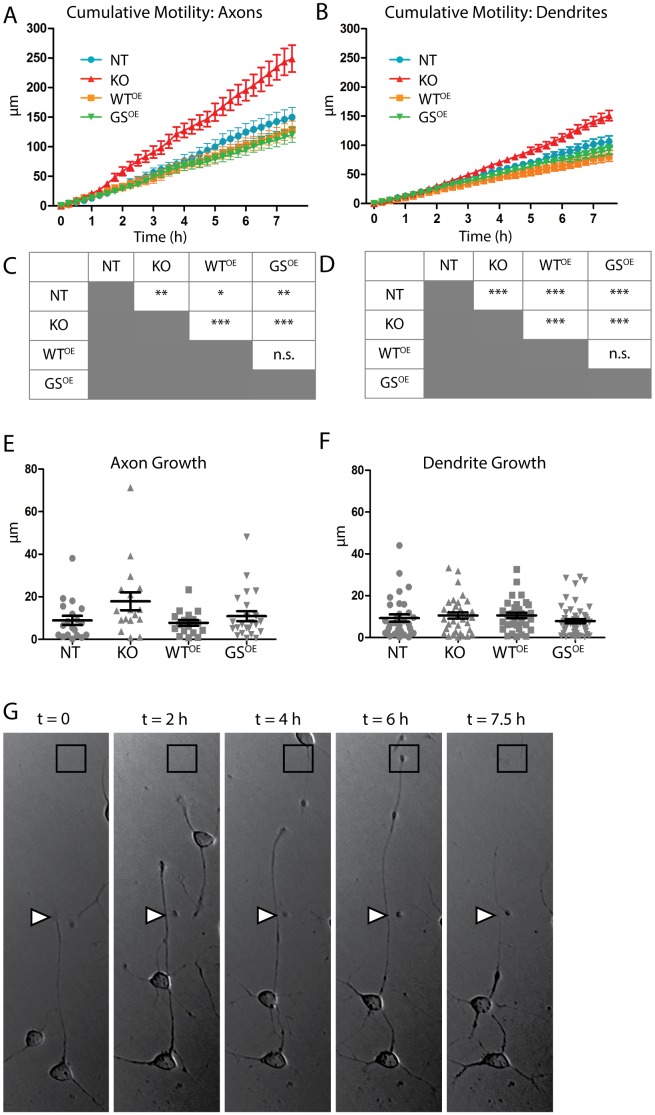
LRRK2-KO neurites show increased motility and those from LRRK2-WT^OE^ and LRRK2-G2019S^OE^ show decreased motility on PLL. Line graphs plot cumulative motility of axons (A) and dendrites (B) over 7.5 hours. Data shown are mean ± SEM. A one-way ANOVA indicates significant differences in motility for both axons and dendrites (see text) and the sources for those differences were identified using Bonferroni's post-tests, and are shown in tables (C) and (D) for axons and dendrites, respectively; *p<0.05; **p<0.01; ***p<0.001. Scatter plots of mean total growth (extension minus retraction ± SEM) of axons (E) and dendrites (F) at the end of imaging. For each genotype, 10–18 neurons were tracked from at least two different culture preparations, one axon per neuron and 2–4 dendritic endpoints. (G) Bright field images of axon extension over time in a KO neuron illustrate the highly dynamic extension and retraction observed. Times (t in h) are indicated at the top. Arrowheads mark the position of the axonal growth cone at t = 0. Squares mark the site of furthest extension of the axonal growth cone, which was observed at t = 6 h.

As expected, axonal growth cones were highly motile, and axons from all genotypes showed periods of extension and retraction throughout the recording period. KO axons displayed significantly increased motility compared to other genotypes. LRRK2-G2019S^OE^ and LRRK2-WT^OE^ axons showed significantly decreased motility relative to NT as well as to KO (Fig. 4A, C). Comparisons of the motility of dendritic growth cones yielded similar results (Fig. 4B, D).

While motility measurements reveal strong differences between genotypes, these differences did not translate ultimately into significant changes in either axon or dendrite growth over the time recorded (Fig. 4E, F; one-way ANOVA; axons, p = 0.054; dendrites, p = 0.172). This is illustrated in Fig. 4G, which shows the initial length of a KO axon (white triangle), steady growth to a maximum at 6 h (black square), followed by retraction over the subsequent 1.5 h, almost returning to its starting length. Together these data indicate that the genotypes examined influence the pattern of growth more robustly than the distance covered.

## Discussion

Here our data show that LRRK2 regulates axonal and dendritic growth and arborization, but the effects are varied and depend on the substrate on which neurons are grown, neuron age, and whether axons or dendrites are examined. Population studies of fixed neurons grown on laminin carried out over the course of three weeks of development reveal a sustained, negative effect of LRRK2-G2019S^OE^ on dendritic growth and arborization, a finding consistent with previous work [12], [24]. Unexpectedly, our findings also reveal a delayed, negative effect of the LRRK2 deletion on dendritic arborization between the second and third weeks *in vitro*. Imaging neurons grown on PLL over several hours reveals reduced motility in neurons overexpressing either LRRK2-WT^OE^ or LRRK2-G2019S^OE^ and heightened motility in neurons lacking LRRK2. Together, our findings support some previous work showing effects of LRRK2 on neurite outgrowth, but they also indicate that neurite growth does not follow a simple correlation with LRRK2 levels and/or mutation, and that outcomes can vary based on the substrate on which neurons are grown and their stage of development.

Several studies have shown that overexpression of LRRK2-G2019S in hippocampal neurons grown in culture [8], [9], [11], in midbrain neurons in culture [12], or in newly born dentate gyrus granule cells *in vivo* [24] can result in shorter neurites. Consistent with these findings, our data show decreased dendritic growth in LRRK2-G2019S^OE^ neurons cultured on laminin. This effect appears to be due to the mutation, and perhaps the increased kinase activity associated with it [4], [6], [26], rather than overexpression, since dendrites in LRRK2-WT^OE^ neurons extend and arborize to a similar extent as dendrites in NT neurons. In LRRK2-G2019S^OE^ neurons, decreased dendritic growth was observed over the entire time course relative to the other genotypes, but the mean differences in growth between genotypes were not dramatic. However, the growth and elaboration of dendritic branches in LRRK2-G2019S^OE^ neurons show consistent upward slopes, suggesting an eventual recovery. This would be consistent with the absence of overt morphological differences that have been reported in a variety of brain areas in adult LRRK2 mouse models [13], [27], [28], [29], but a developmental delay may be relevant to PD as it could impart vulnerability consistent with the adult onset of PD.

The idea that LRRK2 regulates neurite growth has been based on gain-of-function experiments, in which overexpression of human LRRK2-G2019S produces shorter neurites [9], [10], [11], [12], [24] as well as on loss-of-function experiments in which neurons cultured from LRRK2-KO mice produce longer neurites [9], [10]. In the current study, the absence of a consistent effect of LRRK2 deletion on either axon or dendrite extension in neurons grown on laminin is likely due to a ceiling effect generated by maximal growth rates achieved by KO and NT neurons. Our experiments carried out on PLL (a slow-growth substrate) lend credence to this interpretation as they show that axons and dendrites extending from KO neurons exhibit significantly greater motility than those from other genotypes. While motility (a measure that encompasses both extension and retraction) did not translate into increased length over 8 h of imaging, axons showed a trend in this direction. By contrast, LRRK2-G2019S^OE^ neurons showed reduced motility on PLL. However, unlike the long-term studies, where LRRK2-G2019S^OE^ neurons stand apart, LRRK2-WT^OE^ neurons also showed reduced motility suggesting that increased levels of LRRK2, rather than the LRRK2-G2019S mutation, are responsible.

To confirm the substrate dependence of our findings we carried out time lapse imaging of neurons cultured on laminin, but extensive cell body migration, consistent with *in vivo* studies [30], served to confound the time lapse recordings (data not shown). Conversely, we cultured neurons on PLL at low density for long-term studies, but found that postnatal mouse neurons had highly variable survival rates on this substrate, limiting their usefulness. Nevertheless, data from the two independent approaches collectively support that LRRK2 has modest, but consistent effects on the pattern of neurite extension with G2019S^OE^ generally reducing motility and dendrite growth and LRRK2 deletion generally increasing motility. How changes in motility translate into length depends on the environment in which neurons are growing. In vivo, it might be anticipated that axonal extension along tracts or areas enriched with laminin or other growth promoting substrates would be less affected by LRRK2 than the growth of terminal arbors or dendrites within neural parenchyma.

Consistent developmental growth defects seen in culture are likely to have lasting consequences *in vivo*. While this has not yet been investigated, there are some data that support that dendritic arborization in vivo can be altered by LRRK2. Reconstructions of GFP-labeled neurons show decreased dendrite length and branching in newly generated, adult-born dentate gyrus granule cells overexpressing human LRRK2-G2019S compared to non-transgenic controls [24], and in a related study, doublecortin immunolabeling suggests that dendrites may be more elaborate in LRRK2-KO compared to non-transgenic controls [31]. Whether the changes observed are sustained over time is not known.

The effects reported here are less dramatic than might be anticipated based on previous work [9], [10], [11], [12], [24]. This is most likely due to differences in growth substrates as described above, but may also be related to differences in genetic models. Previous studies assessing changes in neurite length used overexpression of human rather than mouse LRRK2 [10], [11], [12], [24]. Thus, it remains possible that there is something inherently different about the human LRRK2 protein or that some observations result from the overexpression of an extremely large (286 kDa) exogenous protein. Human BAC clones may partly mitigate effects of overexpression, but it is not clear whether human LRRK2 regulatory elements are recognized and regulated in mouse the same way as mouse LRRK2.

How LRRK2 regulates growth is not known. Moesin, a member of the ERM (Ezrin, Radixin, Moesin) family has been identified as a putative LRRK2 target [32] and increased LRRK2 activity correlates with increased ERM phosphorylation and decreased axon extension [10]. ERMs cap actin filaments [33], [34], regulate surface expression of cell adhesion proteins and guidance cues [33], [35], and alter axon branching [35], [36], making them especially appealing as LRRK2 effectors for changing patterns of neurite extension. However, LRRK2 has been posited to phosphorylate additional targets that might be expected to alter growth, such as Futsch, a MAP1b ortholog [37]. In addition, certain forms of LRRK2 can generate filamentous structures that decorate microtubules and presumably alter their dynamics [7], [38], [39]. The effects of LRRK2 on both axons and dendrites suggest that either a substrate common to both may be involved, or that some effects may lie downstream of more general effects on cell metabolism [6], [9], [25], [26]. In either case, systematic investigations of LRRK2 substrates will likely prove useful for identifying the relevant signaling pathways.

In summary, our data show that long-term genetic manipulations of LRRK2 expression can produce altered patterns of axonal and dendritic growth, but the effects observed vary with the substrate used. In neurons grown on laminin, LRRK2-G2019S^OE^ slows dendritic growth, but has no effect on the development of polarity or axon growth and arborization. Also on laminin, neurons lacking LRRK2 show normal dendritic growth and arborization through the first two weeks of development after which dendritic arbors show a reduction in terminal branching. On PLL, time lapse imaging shows that KO neurons have more motile axonal and dendritic growth cones compared to NT neurons while LRRK2-G2019S^OE^ and LRRK2-WT^OE^ neurons are less motile than NT neurons. Collectively these data support the idea that LRRK2 can regulate patterns of neurite growth–a finding that may translate into a developmental delay or modest alterations in connectivity. Even a modestly altered course of development could impart vulnerability to environmental influences providing a substrate for a “two-hit” model of PD.

## Methods

### Mouse Models

Mice overexpressing mouse wildtype LRRK2 (LRRK2-WT^OE^) or mouse LRRK2-G2019S (LRRK2-G2019S^OE^) using endogenous promoters were developed as previously described [13]. LRRK2 knockout (KO) mice were generated by the Huaibin Cai lab (National Institutes of Health, Bethesda, MD) [10] and obtained from Jackson Laboratories. Non-transgenic (NT) C57/Bl6 mice were obtained from Charles River Laboratories or from KO littermates. All of the mutant strains used were congenic on an inbred background and backcrossed to their original background.

Mice were housed in the Center for Comparative Medicine at the Mount Sinai School of Medicine. All use of animals was carried out according to protocols that were approved by, and conformed to the guidelines established by, Mount Sinai's Institutional Animal Care and Use Committee and those of the National Institutes of Health.

Postnatal day 1 (P1) mice were anesthetized by hypothermia and quickly decapitated. Hippocampi were dissected and cells dissociated in 0.1% papain, 2.5 mM HEPES (Invitrogen) and 0.1% trypsin inhibitor solution (pH = 7.7). The hippocampal neurons were plated on cover slips coated with poly-L-lysine (PLL; 1 mg/ml; Sigma) alone for 1 day *in vitro* (DIV) time lapse imaging or PLL followed by laminin (13 µg/ml; Sigma) for all other assays. Cells were plated at a density of 2.0×10^5^ cells per plate (4.0×10^4^ cells per cover slip; 1.4×10^4^ cells/cm^2^). Neurons were allowed to adhere for 3 h in Minimum Essential Media (Invitrogen) containing 10% fetal bovine serum (Invitrogen) and 15% sucrose and then transferred to Neurobasal Media (Invitrogen) containing NS21 [40] in a 5% CO_2_, 37°C incubator until live imaging or fixation.

### Immunocytochemistry

Cultured neurons were fixed in 4% paraformaldehyde/4% sucrose for 15 minutes at room temperature (RT) and permeabilized with 0.25% Triton X-100 (Sigma) for 5 minutes at RT. Nonspecific binding was blocked by incubation in 10% bovine serum albumin (BSA) (Sigma) for 1 h at RT. Primary antibodies were diluted in 1% BSA to the appropriate concentration and incubated for 16 h at 4°C. The primary antibodies used include: anti-rabbit polyclonal MAP2 (1∶5000, Abcam, Cambridge, MA) and anti-mouse monoclonal SMI-31 (1∶5000, Abcam, Cambridge, MA). After washing, fluorophore-conjugated secondary antibodies were added for 1 h at RT: anti-mouse DyLight 488 (1∶1200), anti-rabbit DyLight 488 (1∶1200), anti-mouse DyLight 649 (1∶1200), anti-mouse Cy3 (1∶400) (all from Jackson Immunoresearch). Images were acquired on a Zeiss 510 confocal laser scanning microscope.

### Neuronal polarity

Neurons of each phenotype fixed at 3 DIV were labeled for MAP2 and SMI-31 to identify dendrites and axons, respectively. Fluorescence intensities were measured using MetaMorph (Molecular Devices, Sunnyvale, CA). Intensity levels along three 25-pixel-width line segments (1024×1024 pixel-images) were assessed and averaged for both MAP2 and SMI-31 immunofluorescence in one dendrite and in the axon of each neuron. Axonal polarity indices were determined by SMI-31 intensity in dendrites divided by SMI-31 fluorescence in axons (SMI-31_D/A_). Dendritic polarity indices were determined by corresponding quotients of MAP2 intensities (MAP2_D/A_).

### Axon and dendrite outgrowth of fixed neurons

Axons were traced using SMI-31 labeling at 3 DIV neurons using Neurolucida (MBF Bioscience, Williston, VT) to determine length and branching. Dendrites were traced using MAP2 labeling in neurons fixed at 3, 5, 7, 10, 14 and 21 DIV to compare the number of dendrites per neuron as well as the total dendrite length and dendrite branching between neurons of the different genotypes at these ages.

### Statistics

Statistical comparisons between groups were made using Prism 5.0 (GraphPad Software, La Jolla, CA). One-way ANOVA was used for comparisons between genotypes at a single time point and two-way ANOVA was used for comparisons between genotypes over time. For ANOVAs yielding significant differences, the source(s) of difference was determined using Bonferroni's post hoc test. The number of neurons that needed to be traced for the axon and dendrite analyses was estimated by a power analysis (G*Power 3, University of Duesseldorf, Duesseldorf, Germany), which indicated that for a small-sized effect (f = 0.10) between four different groups (genotypes) to be detected at a power of 0.95, at least 20 measurements were needed per genotype. Sample sizes for given experiments are provided in the figure legends.

### Time lapse imaging

Neurons were cultured in a 12-well tissue culture plate coated with PLL alone at a density of 4×10^4^ per well in Neurobasal + NS21 for 24 hours at 37°C and 5% CO_2_. Neurons were imaged for 7.5 hours at 15-minute intervals in a temperature- and CO_2_-controlled chamber, using a Zeiss AxioObserver Z1 with a 20× objective. Axons were defined as the longest process by a difference of at least 10 µm. Axons and dendrites were traced using MetaMorph.
